# “Calcitriol” Is Not Synonymous with “Vitamin D”

**DOI:** 10.1155/2012/650462

**Published:** 2012-10-23

**Authors:** Samantha M. Kimball, Heather E. Hanwell

**Affiliations:** ^1^Department of Chemistry and Chemical Engineering, Royal Military College of Canada and Department of Nutritional Sciences, University of Toronto, Toronto, ON, Canada M5S 3E2; ^2^Neurosciences and Mental Health, Hospital for Sick Children, University of Toronto, Toronto, ON, Canada M5G 1X8

Regarding the recent article by Shaygannejad et al. [1], we are writing to express serious concerns about the portrayal of the intervention and the authors' interpretation and discussion of the findings. Of predominate concern is that the authors utilized a calcitriol intervention in their study but referred to it as “vitamin D”. The erroneous use of the term “vitamin D” is of concern for several reasons.

Firstly, vitamin D is the biologically inactive form that can be synthesized in the skin when exposed to ultraviolet B radiation and is found in foods. In order to produce the active metabolite, calcitriol (or 1,25-dihydroxyvitamin D; 1,25(OH)_2_D), vitamin D must be hydroxylated in two subsequent reactions by two separate hydroxylases. The second hydroxylation step—conversion of 25-hydroxyvitamin D [25(OH)D] to 1,25(OH)_2_D via 1*α*-hydroxylase—is under tight regulatory control. By providing adequate substrate (i.e., vitamin D), circulating 25(OH)D concentrations increase and cells are able to locally produce and use 1,25(OH)_2_D in a self-regulated manner without affecting circulating levels of calcitriol. In contrast, supplying calcitriol directly produces a systemic increase in calcitriol, thus bypassing the key regulatory steps and increases risk of hypercalcemia. On the other hand, supplementation with vitamin D is quite safe at levels up to 10,000 IU/d [2, 3]. Wingerchuk et al. [4] have previously demonstrated the risk of hypercalcemia with calcitriol treatment in patients with MS. With this sole exception [4], none of the published vitamin D-related trials in patients with MS have administered calcitriol; the authors failed to note this or compare the findings of their calcitriol intervention to the only other published calcitriol intervention in MS. The differences in the safety profile and activity of vitamin D versus calcitriol alone are significant and necessitate accurate nomenclature.

Next, the authors indicate “no unusual or unexpected safety risks found with vitamin D therapy in our study population with RRMS”; however, not only did they administer calcitriol rather than vitamin D, but also no characterization of the dosage nor description of the rationale for the selected dosage was present. Furthermore, since there is neither mention of serum calcium, urine calcium nor 1,25(OH)_2_D levels in response to therapy dose, we cannot assess the effect of oral calcitriol [1,25(OH)_2_D] supplementation on biochemical outcomes in these patients.

Overall, the authors' naivety regarding metabolism of vitamin D is evidenced in that they (i) neglected to monitor calcium or calcitriol levels when treating with calcitriol, (ii) monitored 25(OH)D levels—which are the biomarker of vitamin D status in the normal situation but are not relevant during calcitriol therapy, and (iii) directly compare studies of supplementation with vitamin D, calcitriol, and alphacalcidiol (a synthetic analogue of calcitriol) without distinguishing the various forms and their inherently different activity.

In conclusion, the work presented here “*a calcitriol intervention in adults with MS*” cannot be directly compared with the majority of the previous vitamin D-related MS interventions in the literature. Barring instances of genetic mutations causing dysfunction of the 1-alpha hydroxylase, at present, there is no rationale to support the use of calcitriol over vitamin D.
